# Factors associated with sexual dysfunction in Taiwanese females with rheumatoid arthritis

**DOI:** 10.1186/s12905-017-0363-5

**Published:** 2017-02-14

**Authors:** Miao-Chiu Lin, Ming-Chi Lu, Hanoch Livneh, Ning-Sheng Lai, How-Ran Guo, Tzung-Yi Tsai

**Affiliations:** 1 Department of Nursing, Dalin Tzuchi Hospital, The Buddhist Tzuchi Medical Foundation, 2 Minsheng Road, Dalin Township, Chiayi, 62247 Taiwan; 2 Division of Allergy, Immunology and Rheumatology, Dalin Tzuchi Hospital, The Buddhist Tzuchi Medical Foundation, 2 Minsheng Road, Dalin Township, Chiayi, 62247 Taiwan; 3 School of Medicine, Tzu Chi University, 701 Jhongyang Road Section 3, Hualien, 97004 Taiwan; 4 Rehabilitation Counseling Program, Portland State University, Portland, OR 97207-0751 USA; 5 Department of Occupational and Environmental Medicine, National Cheng Kung University Hospital, 138 Sheng-Li Road, Tainan, 70428 Taiwan; 6 Occupational Safety, Health, and Medicine Research Center, National Cheng Kung University, 138 Sheng-Li Road, Tainan, 70428 Taiwan; 7 Department of Environmental and Occupational Health, College of Medicine, National Cheng Kung University, 138 Sheng-Li Road, Tainan, 70428 Taiwan; 8 Department of Medical Research, Dalin Tzuchi Hospital, The Buddhist Tzuchi Medical Foundation, 2 Minsheng Rd., Dalin Township, Chiayi, 62247 Taiwan; 9 Department of Nursing, Tzu Chi University of Science and Technology, 880 Chien-Kuo Road Section 2, Hualien, 62247 Taiwan

**Keywords:** Sexual dysfunction, Female, Rheumatoid arthritis, Taiwan

## Abstract

**Background:**

Patients with rheumatoid arthritis (RA) may experience sexual dysfunction because of symptoms or adverse effects from treatments. Data on female sexual dysfunction (FSD) in Asian females with RA issue are limited. This study investigated the prevalence and factors associated with FSD in Taiwanese patients with RA.

**Methods:**

This cross-sectional study used a purposive sampling method to recruit 195 females with RA from a single hospital in southern Taiwan. Demographic and clinical characteristics were obtained by review of medical records and a structured questionnaire. The Chinese version of the Female Sexual Function Index and the Taiwanese Depression Questionnaire were also administered. Multiple logistic regression analysis was used to identify factors associated with FSD.

**Results:**

The crude and age-standardized prevalence of FSD were 66.8% and 48.2%, respectively. Patients who were older, with a comorbid condition, with more depressive symptoms, and with greater disease activity had a significantly higher risk of FSD.

**Conclusion:**

Our findings indicate that FSD is more common in Taiwanese individuals with RA who have certain specific demographic and clinical characteristics. These findings may help to identify and facilitate the provision of appropriate interventions to ensure better sexual health in female patients with RA.

## Background

Rheumatoid arthritis (RA) is a systemic autoimmune disease characterized by inflammation and progressive damage of the joints that affects 0.5–1.0% of the population worldwide [1]. RA onset usually occurs in individuals who are 30 to 50 years old, and about 20–30% of affected individuals report some arthritis-attributable work limitations, with major burdens to patients, families, and social care systems [2]. Gabriel and colleagues [3] estimated the direct annual costs for care of an RA patient was US$3802 in 1987 (corresponding to US$5763 in 2000), approximately six-times higher than for an individual without RA. Additionally, the annual total societal costs (sum of direct, indirect, and intangible costs) was estimated to exceed US$39 billion [4].

There have been massive increases in specialized diagnostic and therapeutic methods, and this has improved the survival of RA patients in recent decades. However, some treatments may lead to the onset of negative sequelae, such as fatigue, sadness, and physical changes, and these may influence a patient’s sexual function and desire for sexual intercourse. Previous research estimated that about 46% to 75% of females with RA had female sexual dysfunction (FSD) [5–9], which was approximately twice as high as for the healthy women [10]. Notably, most women develop RA between the ages of 30 and 50 years, which is within the age range for pregnancy. Accordingly, the reduced sexuality that accompanies RA leads to deterioration in quality of life (QOL) and family function, and may also result in divorce [5]. In view of this, eliminating FSD in RA patients has become a primary priority in healthcare practice [11, 12].

Most studies of FSD in individuals with RA have been conducted in Western countries [5, 8, 9, 13, 14]. Due to the more conservative Asian culture, Chinese people often regard sex as a taboo subject and are more reluctant to openly talk about their sex lives [15]. Thus, a review of the literature indicated that most studies of RA in Taiwan have focused on the effects of medical therapy [16], ambulatory care utilization [17], and disease epidemiology [18]. There is very little known about FSD in Chinese individuals with RA. So this study aimed to examine the prevalence and factors associated with FSD in RA patients from Taiwan. The findings of this study could serve as a reference for the recognition of FSD in Chinese individuals with RA as well as may be useful for implementation of interventions.

## Methods

### Study design and population

This is a cross-sectional study of female outpatients and inpatients with RA who were recruited consecutively from July 2014 to June 2015 at a single hospital in southern Taiwan (Dalin Tzuchi Hospital, Chiayi, Taiwan). The inclusion criteria were as follows: *(i)* aged 20 years or older; *(ii)* no cognitive impairments and with the ability to express opinions in Mandarin or Taiwanese; *(iii)* sexually active for at least 2 years before the diagnosis of RA; and *(iv)* awareness of the diagnosis of RA and agreement to participate in the survey. The sample size needed for this study was determined as described by Cohen [19]. For an α of 0.05, power of 0.8, and effect size of 0.15, this analysis indicated the need for a sample size of at least 150 patients.

### Instruments

Three measures were used to survey the enrolled patients: the Taiwanese Depression Questionnaire (TDQ), the Female Sexual Functional Index (FSFI), and a questionnaire that requested information on demographic variables and clinical characteristics.

To assess the presence of depressive symptoms, we administered the TDQ which was created by Lee et al. and was developed specifically to meet the needs of the Asian culture [20]. The test is comprised of 18 items, each of which assesses symptoms during the past one week, using a scale of 0 (absence of symptoms) to 3 (presence of symptoms almost every day). The total score therefore ranges from 0 (no depression) to 54 (significant depression). Based on comparison with the Structured Clinical Interview for DSM Disorders (SCID) as the gold standard, the TDQ had good concurrent validity, and the area under the receiver operating characteristic (ROC) curve was 0.92. The TDQ performed optimally using a cutoff value of 19 in detecting depressive symptoms in patients with chronic diseases or from the general population [21, 22]. Assessment of test reliability indicated that the TDQ had good internal consistency among different groups of subjects, and Cronbach’s α ranged from 0.89 to 0.92 [21–23]. Cronbach’s α from the present data was 0.91.

The Female Sexual Function Index (FSFI), developed by Rosen and colleagues [24], was used to measure FSD. This 19-item questionnaire was developed as a brief, multidimensional self-reporting instrument to assess the key dimensions of sexual function over the previous four weeks in six domains: desire, subjective arousal, lubrication, orgasm, satisfaction, and pain. The total score was obtained by adding the six separate domain scores, and ranged from 2.0 to 36.0. A lower score indicated more severe FSD. Previous studies have evaluated the FSFI for discriminant validity, divergent validity, concurrent validity, and test-retest reliability [24–26]. In clinical practice, an FSFI cut-off score of 26.55 has been widely used to define FSD [15, 27]. This test was translated into Chinese by Kuo et al., and Cronbach’s α was 0.81 to 0.92 for all domains in the Chinese version [28]. Cronbach’s α from the present study yield a coefficient of 0.91.

We also used questionnaires that assessed demographic and clinical characteristics that were based on a review of previous literature and clinical experience. The demographic data included age, marital status, educational level, job status, living status, religious beliefs, and certain lifestyle factors such as smoking and exercise habits. Those who answered “currently” or “yes/past” to smoking were classified as smokers. Those who exercised 3 or more days per week were classified as having regular exercise habits. The clinical characteristics included the following: chronic disease (diabetes mellitus, hypertension, heart disease, or stroke), body mass index (BMI), Disease Activity Score in 28 Joints (DAS28), serum C-reactive protein (CRP), duration of RA, menopausal status (premenopause or postmenopause), depressive symptoms, self-reported pain based on a visual analog scale (VAS), and use of biological disease-modifying anti-rheumatic drugs (DMARDs), such as Etanercept, Adalimumab, Infliximab, or Rituximab. For this last variable, participants were asked whether they had ever used these biological DMARDs for more than 3 months after RA onset. All clinical characteristics were obtained by chart review.

### Data collection

This study was approved by the Institutional Review Board of Dalin Tzuchi Hospital. Before enrolling in the study, all participants received detailed written and verbal information regarding the aims and protocol of the study and signed an informed consent. The researchers were available to answer any inquiries during completion of the questionnaires. For illiterate patients, the researchers read the questionnaires and recorded answers. All questionnaires were returned without any identifying personal information and were only marked with an encryption code to facilitate data analysis. The encryption rules were available for the researchers only.

### Statistical analysis

Descriptive and inferential statistical analyses were conducted in accordance with the study aims and the nature of variables. Descriptive statistics (mean and standard deviation [SD]) were used to describe the demographic and clinical characteristics. For inferential analysis, a *t*-test or chi-square test was used to identify the relationships of demographic and clinical characteristics with FSD (cut off score of 26.55). Variables significantly related to FSD in the univariate analysis were entered into a multiple logistic regression to compute adjusted odds ratios (aORs) and 95% confidence intervals (CIs). The α value was set at 0.05 for all statistical analyses.

## Results

### Demographic and clinical characteristics of participants

During the recruitment period, we approached 195 women with RA. Among them, 131 met the criteria for FSD based on an FSFI score of 26.55 or less (crude prevalence: 66.8%). After adjusting for age based on the 2000 World Standard Population [29], the age-standardized prevalence of FSD was 48.2%. Thus, about half of the individuals with RA in this sample suffered from FSD.

The mean age of participants was 53.76 years old (±8.89), and most of them were married (92.9%), unemployed (55.1%), cohabitating (92.9%), and with a high level of education (55.1%). In addition, most participants had religious beliefs (85.2%), did not smoke (95.4%), engaged in regular exercise (67.9%), and were in menopausal status (63.8%). The mean duration of RA was 9.72 years (±6.02). Nearly 70% of the participants reported use of a biological DMARD, and 44.4% had a comorbid condition. The overall mean BMI, DAS28 score, serum CRP level, pain score, and TDQ score were 24.00, 3.77, 0.94, 3.18, and 12.28, respectively (Table 1).

**Table 1:** Demographic and clinical characteristics of enrolled Taiwanese females with RA (n = 196)

Variable	Mean ± SD	N (%)
Demographic characteristics		
Educational level		
High (≥9^th^ grade)		108 (55.1)
Low (<9^th^ grade)		88 (44.9)
Martial status		
Married		182 (92.9)
Single		14 (7.1)
Working status		
Employed		88 (44.9)
Unemployed		108 (55.1)
Living status		
Living alone		14 (7.1)
Cohabitating		182 (92.9)
Religious beliefs		
Yes		167 (85.2)
No		29 (14.8)
Cigarette smoking		
Yes		9 (4.6)
No		187 (95.4)
Regular exercise		
Yes		133 (67.9)
No		63 (32.1)
Menopausal status		
Yes		125 (63.8)
No		71 (36.2)
Age (years)	53.76 ± 8.89	
Clinical characteristics		
Comorbidity		
Yes		87 (44.4)
No		109 (55.6)
Use of a biological medication for RA		
Yes		131 (66.8)
No		65 (33.2)
Disease duration (years)	9.72 ± 6.02	
BMI (kg/m^2^)	24.00 ± 4.19	
DAS28 score	3.77 ± 1.01	
Serum CRP (mg/dL)	0.94 ± 1.07	
Self-reported pain (VAS)	3.18 ± 3.03	
TDQ score	12.28 ± 8.34	

### FSFI scores

The mean FSFI score was 11.87, with a SD of 7.05. Of the six domains of this index, “satisfaction” was found to reveal the highest standardized score, 54.19, whereas “arousal ” showed the lowest standardized score of 23.8 (Table 2).

**Table 2:** Mean and SD of the six domains of FSFI (n = 196)

Domain	Mean	SD	Standardized score^(1)^	Rank
Desire	1.98	0.97	33.0	2
Arousal	1.43	1.66	23.8	6
Lubrication	1.89	2.23	31.5	3
Orgasm	1.85	2.16	30.8	4
Satisfaction	3.25	1.21	54.2	1
Pain	1.48	1.72	24.7	5

^(1)^Standardized score = mean ÷ total score*100%

### Correlations of demographic and clinical characteristics with FSD

Table 3 shows the demographic and clinical characteristics of participants with and without FSD. This univariate analysis indicates that those with FSD were more likely to be unemployed (*p* = 0.04) and older (*p* < 0.001), have less education (*p* < 0.001), have a comorbidity (*p* = 0.003), and be in menopausal status (*p* < 0.001). Moreover, FSD was more common in women with a longer duration of RA (*p* = 0.04), more severe depressive symptoms (*p* < 0.001), higher DAS 28 score (*p* = 0.003), and more pain (*p* = 0.03).

**Table 3:** Relationship of demographic and clinical characteristics with FSD in Taiwanese RA patients (n = 196)

Variable	FSD (n=131)	Non-FSD (n=65)	*p*
	N (%) or Mean ± SD		
Demographic characteristics			
Educational level			<0.001
High (≥9^th^ grade)	60 (45.8)	48 (73.8)	
Low (<9^th^ grade)	71 (54.2)	17 (26.2)	
Martial status			0.33
Married	120 (91.6)	62 (95.4)	
Single	11 (8.4)	3 (4.6)	
Working status			0.04
Employed	52 (39.7)	36 (55.4)	
Unemployed	79 (60.3)	29 (44.6)	
Living status			0.12
Living alone	12 (9.2)	2 (3.1)	
Cohabitating	119 (90.8)	63 (96.9)	
Religious beliefs			0.15
Yes	115 (87.8)	52 (80.0)	
No	16 (12.2)	13 (20.0)	
Cigarette smoking			0.14
Yes	4 (3.1)	5 (7.7)	
No	127 (96.9)	60 (92.3)	
Regular exercise			0.21
Yes	85 (64.9)	48 (73.8)	
No	46 (35.1)	17 (26.2)	
Menopausal status			<0.001
Yes	96 (73.3)	30 (46.2)	
No	35 (26.7)	36 (55.4)	
Age (years)	56.19 ± 7.16	47.41 ± 8.02	<0.001
Clinical characteristics			
Comorbidity			0.003
Yes	68 (51.9)	19 (29.2)	
No	63 (48.1)	46 (70.8)	
Use of a biological medication for RA			0.43
Yes	90 (68.7)	41 (63.1)	
No	41 (31.3)	24 (36.9)	
Disease duration (years)	10.33 ± 6.12	8.48 ± 5.45	0.04
BMI (kg/m^2^)	24.35 ± 4.34	23.30 ± 3.28	0.10
DAS28 score	3.92 ± 1.06	3.46 ± 0.84	0.003
Serum CRP (mg/dL)	1.05 ± 1.53	0.71 ± 2.17	0.21
Self-reported pain (VAS)	3.48 ± 3.27	2.57 ± 2.39	0.03
TDQ score	13.98 ± 8.79	8.83 ± 6.09	<0.001

### Independent predictors of FSD

Multiple logistic regression analysis indicated that 4 variables were significantly and independently associated with FSD. In particular, FSD was more likely in subjects who had a comorbidity (aOR: 2.76, 95% CI: 1.43–6.94), were older (aOR: 1.22, 95% CI: 1.13–1.33), had a higher TDQ score (aOR: 1.18, 95% CI: 1.08–1.26), and had a higher DAS28 score (aOR: 1.38, 95% CI: 1.15–2.23) (Table 4).

**Table 4:** Multiple logistic regression analysis of the association of demographic and clinical characteristics with FSD in Taiwanese RA patients

Variable	Crude OR (95% CI)	Adjusted OR (95% CI)
Educational level		
Low (<9^th^ grade)	3.34 (1.74-6.40)	1.55 (0.65-3.67)
High (≥9^th^ grade)	1	1
Working status		
Unemployed	1.88 (1.03-3.44)	1.32 (0.60-2.89)
Employed	1	1
Menopause		
Yes	3.40 (1.83-6.35)	2.67 (0.88-7.49)
No	1	1
Comorbidity		
Yes	2.61 (1.39-4.93)	2.76 (1.43-6.94)
No	1	1
Age (years)	1.15 (1.10-1.21)	1.21 (1.13-1.32)
Disease duration (years)	1.06 (1.01-1.12)	1.03 (0.96-1.11)
DAS28 score	1.73 (1.19-2.52)	1.38 (1.15-2.23)
Self-reported pain (VAS)	1.12 (1.00-1.25)	1.01 (0.89-1.14)
TDQ score	1.11 (1.05-1.17)	1.18 (1.08-1.26)

## Discussion

To the best of our knowledge, this is the first study to investigate the prevalence and factors associated with FSD in Chinese individuals with RA. We found that the crude and age-standardized prevalence of FSD in our sample were 66.8% and 48.2%, respectively. These numbers are consistent with those of previous reports, which indicated that the prevalence of FSD in subjects with RA ranged from 45% to 62% [8, 13, 14]. Table 2 further demonstrates that severe FSD is primarily a reflection of the “arousal” and “pain” domains. It may be inferred from these findings that RA patients who have stiffness and joint deformity in their hips or shoulders experience difficulty with intercourse and finding a comfortable sexual position, and this may lead to poor sexual satisfaction [9]. In addition, the types of drugs that patients are being prescribed, such as non-steroidal anti-inflammatory drugs (NSAIDs), have been shown to be related to the sexual arousal [30]. Notwithstanding the fact that FSD is more prevalent in subjects with RA, its detection and management are still not fully recognized as part of the routine care for this population [31]. Therefore, healthcare providers should be informed that not only must they be aware of the occurrence of FSD among RA patients, but they should also actively assess the effects of medications prior to using them in treating the related symptom of RA.

The results of our multivariate analysis indicated that older age correlated positively with FSD, in agreement with previous findings [8, 10, 11, 14]. In general, as females get older, hormone production is lower, and this may cause epithelial atrophy, decline of physical strength, and vaginal dryness, all of which can provoke dyspareunia and decreased libido [32]. Hormone replacement therapy might be recommended for female RA patients who suffer from vaginal atrophy or irregular menstrual cycles. It not only increases vaginal secretion or the vaginal wall elasticity to enjoy the sexual life [32], but also results in an attenuation of serum levels of interleukin 6 (IL-6) receptor, which is related to the biological activity of IL-6 [33]. Some non-pharmacological or noninvasive approaches, such as lubricants and vaginal estrogen creams, may be taken into consideration [34].

Our study revealed that the presence of a chronic medical condition significantly increased the risk of FSD among RA patients, in agreement with previous findings [13, 14]. This may be because the presence of a comorbid condition negatively affects a patient’s perception of her health status, or reduces her ability to withstand therapy-induced side effects and other complications arising from RA. Our results also agree with those of earlier reports which showed a greater risk of FSD among RA patients with depressive symptoms [8, 9, 11, 13]. It is noteworthy that a review article highlighted that some antidepressant medications, especially selective serotonin uptake inhibitors, can have deleterious effects on sexuality because they reduce sexual arousal and vaginal lubrication [35]. Depression is a common psychiatric disorder among individuals with RA [36], so clinicians should carefully appraise the effects of antidepressant medications before prescribing them to these patients. This approach might help to reduce FSD in this population.

Consistent with some previous studies [8, 11–13], we also found a significantly positive correlation between DAS28 score and FSD among individuals with RA. This implies that those with better mobility are more likely to successfully integrate into social support networks, maintain good interpersonal relationships, require less help from others in daily activities, and have better sexual function. This finding, however, differed from some other reports [10, 14]. This inconsistency may be attributed to the differences in age distribution of study subjects or the use of different statistical analyses. For example, our subjects (average age: 53.8 years) were older than those in the study of Costa et al. (49.7 years) [10]. It can be argued that different physical and possibly cognitive functions may have different effects on sexual function. Furthermore, both of these former studies relied solely on univariate analysis [10, 14], and therefore did not account for potential confounding.

The results of the present study should be interpreted with caution because of several limitations. First, all participants were drawn from a single hospital in southern Taiwan, so the results might not be generalizable to other populations. Future studies should recruit larger samples *via* a nationwide survey or random sampling to improve the representativeness of findings. Nonetheless, we calculated the sample size needed to ensure adequate statistical power before beginning this study and obtained some statistically significant findings, so the sample size may be considered sufficient for identifying factors associated with FSD. However, future studies using non-RA subjects as a reference group for comprehensive risk comparison are still recommended. Second, this study used a cross-sectional design so we cannot confirm causality. A longitudinal research design is needed to establish causal relationships, especially focusing on the long-term relationship between FSD and clinical prognosis. Third, although we accounted for the influence of potential confounders, residual confounding might have been present due to unmeasured confounders (such as distress mood, coping effectiveness, diet, or genotype). Future studies are recommended to examine these concerns via the employment of more psychometrically sound scales. Despite these methodological concerns, this might be the first study to assess the prevalence of FSD among individuals with RA in Taiwan. Thus, this study can be used as a reference for the development of timely therapeutic regimens for treatment of FSD in patients with RA.

## Conclusions

Advances in medical techniques have extended the survival and improved the lives of patients with RA, but disease symptoms and the adverse effects of different treatments may affect the sexual health of these patients. This study found that the crude and age-standardized prevalence of FSD among women with RA were 66.8 and 48.2%, respectively. Those who were older, had a comorbid condition, had more depressive symptoms, and had greater disease activity had a higher risk for FSD. Healthcare providers should actively institute appropriate rehabilitation procedures for female RA patients with sexual dysfunctions. Ensuring that sexual health is available to female RA patients may be an important first step to helping them to better cope with their disease and may also help to improve their overall quality of life and survival.

## ᅟ

aOR: Adjusted odds ratio
BMI: Body mass index
CI: Confidence interval
CRP: C-reactive protein
DAS28: Disease Activity Score in 28 Joints
DMARDs: Disease-modifying anti-rheumatic drugs
FSFI: Female Sexual Functional Index
FSD: Female sexual dysfunction
IL-6: Interleukin 6
QOL: Quality of life
RA: Rheumatoid arthritis
ROC: Receiver operating characteristic
SCID: Structured Clinical Interview for DSM Disorders
SD: Standard deviation
TDQ: Taiwanese Depression Questionnaire
VAS: Visual analog scale
